# A Panoramic View of Patients’ Beliefs and Knowledge About Chronic Respiratory Disease

**DOI:** 10.7759/cureus.31633

**Published:** 2022-11-18

**Authors:** Manjulakshmi Padmanabhan, Tamilarasu Kadhiravan, Manju Rajaram, Gitanjali Batmanabane

**Affiliations:** 1 Pharmacology, Jawaharlal Institute of Postgraduate Medical Education & Research, Puducherry, IND; 2 Medicine, Jawaharlal Institute of Postgraduate Medical Education & Research, Puducherry, IND; 3 Pulmonary Medicine, Jawaharlal Institute of Postgraduate Medical Education & Research, Puducherry, IND

**Keywords:** qualitative research, medication adherence, psychological stress, chronic obstructive pulmonary disease, asthma

## Abstract

Objective

Patients with chronic respiratory diseases (CRD) like asthma and chronic obstructive pulmonary disease (COPD) experience significant morbidity and mortality. The patient's quality of life deteriorates with the progression of the disease. Pharmacological treatment focuses on reducing the symptoms. The psychological impact of the disease on the patient's quality of life is not assessed by all healthcare providers. There is limited knowledge about the patient's demographic and clinical factors affecting the quality of life in CRD patients and aspects hindering or influencing the management of disease in this population. Hence we aimed to conduct a qualitative study on patients with asthma or COPD to get a view of their knowledge about the disease, the problems they encounter in their day-to-day life and the treatment.

Methods

Semi-structured, face-to-face interviews were conducted by the investigator in the hospital during the patient's follow-up visits. The questions focused on the patient's awareness of the disease, living with chronic respiratory disease, understanding of disease and treatment, and compliance with inhaled therapy. The interviews were audio recorded and transcribed verbatim. Content analysis of the data was done manually. Codes and themes were derived manually. Themes were formed from the codes and sub-themes.

Results

Five themes were generated from the data obtained: (1) misconceptions regarding the contagious nature of the disease; (2) psychological stress due to feelings of worthlessness, helplessness due to inability to work and poor understanding among family members; (3) inappropriate lifestyle modifications like avoiding fruits and vegetables due to the fear of acute attacks; (4) poor adherence to inhalers due to work timing and difficulty travelling; and (5) lack of reinforcement by the healthcare providers on inhaler technique and adherence were identified as causes of poor inhaler technique and inappropriate knowledge about drugs.

Conclusion

Subjective reporting by patients in this study was helpful in understanding issues concerning disease management in CRD patients. Apart from assessing the patients’ symptoms and prescribing drugs, healthcare providers should take time to impart knowledge about the disease to patients. Though patient education and psychological intervention are challenging to implement daily, they are supplemental to the pharmacologic management of the disease.

## Introduction

Asthma and chronic obstructive pulmonary disease (COPD) are common chronic respiratory diseases (CRD) which cause significant morbidity and mortality. In India, the disease burden is significantly high due to under-diagnosis, undertreatment, increasing pollution and the use of biomass fuel [1]. In India, 3% of adults are affected by asthma, according to a multicentric study [2] and 6.5-7.7% are affected by COPD [3]. In 2015, asthma caused 1.1% of global disability-adjusted life years (DALYs), and COPD caused 2.6% of global DALYs [3]. The burden of the disease is intense during the episodes of acute exacerbations [4]. The disease also produces significant psychological stress, affecting disease control. CRD patients experience more psychological problems and have worse social functioning than other average members of the community [5].

The primary aim of treatment is to attain disease control. Addressing the physiological symptoms of the disease using pharmacological treatment focuses on reducing symptoms. This does not guarantee complete disease control. The psychological stress experienced by patients needs to be studied and addressed to attain disease control. Another aspect to be acknowledged while managing chronic diseases is the quality of life experienced by the patients [6].

Not all healthcare providers assess the psychological impact of the disease on the patient's life. The psychological stress perceived by the patient depends on different factors, namely individual-level factors like socioeconomic status, family-level factors like maternal and paternal stress, and community-level factors like air pollution and housing [7]. There is no appropriate estimate of the psychological impact of the disease on the patient's life or disease control [8]. Qualitative research provides a valuable description of the medical, social and emotional problems encountered by patients with CRD on inhaler treatment.

This study was carried out in an institution where patients receive treatment and drugs free of cost. This government policy was implemented to reduce the financial burden on the patients. The majority of the patients visiting this hospital have no formal education, belong to low socioeconomic status and live in rural areas as per the previous study [9]. With the monetary issues being taken care of, the other psychological impacts of the disease on their life needs to be studied. There is limited knowledge about the patient's demographic and clinical factors affecting the quality of life in CRD patients and aspects hindering or influencing the management of disease in this population. Hence the aim was to conduct a qualitative study on patients with asthma or COPD to get an insight into their knowledge about the disease, the problems encountered during their day-to-day life and the treatment to improve disease control.

## Materials and methods

Study setting

This study was conducted in a tertiary care hospital in Puducherry, South India. Institute ethics committee approval was obtained (JIP/IEC/2015/15/571 dated 30-06-2015). Patients between the age group 14 and 80 years with an established diagnosis of either asthma or COPD, with or without other comorbid diseases, who have been using inhalers for at least three months, were eligible to participate in the study. Patients who are contraindicated to performing pulmonary function test (PFT), pregnant women, and patients not willing to participate in the interview were excluded. The interview was carried out on 32 patients, beyond which data saturation was attained. Written informed consent was obtained from the patient.

Data collection

The patients’ sociodemographic details were obtained. The duration of disease and inhaler use technique was noted. The disease severity was assessed using the asthma control test (ACT) and COPD assessment test (CAT) questionnaires for asthma and COPD, respectively. PFT (NSpire HDpft 4000, Pune, India) was done for all patients. All patients provided consent, and interviews were not repeated.

Study design

Patients were explained about the study, and consent was obtained for the interview audio recording. This is a phenomenological study. In-depth, semi-structured, face-to-face interviews were conducted. The topics and semi-structured, open-ended questions to address these topics were prepared based on previous literature (Table 1) [10, 11,12].

**Table 1:** Topics of discussion and interview guide for patients with chronic respiratory disease

Topics of discussion	Interview-guide
Patient's awareness of their disease	What do you call your disease?
	Do you know the nature of your disease?
	Why do you think it started?
	What do you think caused the problem?
Severity of their disease	What do you think the sickness does?
	Describe your symptoms
	How severe is the sickness?
	What are the main problems your disease has caused?
	What aspect of the illness worries you the most?
Coping measures and the need for lifestyle modifications	How is it to live with your disease?.
	How do you cope with your disease?
	Is there a need for any lifestyle modifications? If yes, please elaborate
Understanding inhaler treatment and difficulties faced while using them	How comfortable are you with the inhaler use?
	Are you using the inhaler as indicated? If not why.
Adherence issues	Are you compliant with the inhaled medication?
	If not, why do you miss the dose?
Other concerns	Any other areas of interest or concern to patients. Exploring specific issues or queries that had previously been identified or had arisen earlier in the discussion.

The first author, a female PhD scholar trained in qualitative research methods, conducted the interviews. The author introduced herself to the patients and primed regarding the interview during their follow-up visit with the physician. The interview was conducted at a time feasible for the patient in the hospital.

No person other than the investigator and patient was present during the interview. If interested, family members accompanying the patient were allowed to participate in the interview. No questions were framed from the perspective of the accompanying person, but they were allowed to voice their comments related to the above areas during the interview. The duration of the interview ranged from 20-30 minutes. The interviews were audio recorded and transcribed verbatim. The patient’s identity was not revealed. Content analysis of the data was done manually. Codes and themes were derived manually. The data were coded by two coders, and uniformity was reached by discussion. Themes were formed from the codes and sub-themes.

## Results

Patient characteristics

A total of 32 patients participated in this qualitative study. Of which 23 were asthma patients and nine were COPD patients. The baseline characteristics of the study population show the mean age of the study group was 42 years (Table 2). Based on the forced expiratory volume at 1 second (FEV1) values, out of the 23 asthma patients, eight had mild, 10 had moderate, and five had severe disease. According to ACT scores, 10 had controlled, seven had partially controlled, and six had uncontrolled disease. All nine COPD patients had severe disease based on CAT scores. Six COPD patients had severe disease, one with moderate and two with mild disease states based on FEV1 values.

**Table 2:** Baseline patient characteristics ACT-asthma control test; CAT-COPD assessment test; FEV1-forced expiratory volume at 1 second; SD-standard deviation; yrs-years; COPD-chronic obstructive pulmonary disease

Variable	Total	Asthma (n=23)	COPD (n=9)
Male (n)	13	6	7
Female (n)	19	17	2
Age (yrs) (mean±SD)	42.3±12.6	37.3±12.9	55±13.1
Duration of disease (yrs) (mean±SD)	10.4±8	8.6±7.3	15±7.8
Duration of inhaler use (yrs) (mean±SD)	4.2±3.3	4.2±3.3	4.3±3
ACT/CAT score (mean±SD)	-	19.4	26
FEV1 (%) (mean±SD)	64.2±25.6	68±24.5	54.7±25.8

Qualitative analysis

The data generated from the interview were coded, and five themes were developed. They were (1) misconception about their disease, (2) psychological stress due to the disease, (3) irrelevant lifestyle modifications, (4) poor adherence, and (5) lack of reinforcement.

Theme 1: Misconception about the disease

Almost all patients were ignorant about the cause and chronic nature of the disease. The patients referred to their disease condition as “breathlessness”, “cold symptoms”, or “breathing problem” with chest tightness and cough. Patients would identify their disease condition with the symptoms and are unaware that they are the manifestations of the disease.

Two patients with asthma diagnosed about a year back assumed that doctors would stop the medicines as they were free of symptoms for the past few months. These patients were shocked and annoyed when told they needed to take their medications for their lifetime to keep the disease under control (Table 3). This displays ignorance among patients about their condition and drug. Family members of asthma patients misjudged the patient’s cough to be contagious. Older patients were isolated from their family members, especially grandchildren, for fear of spreading the disease. Patients strongly believed there is an association between developing asthma and the ingestion of fruits, certain vegetables and dairy products. They also feared consuming these foods might exacerbate their symptoms. A fallacy among most patients regarding addiction to inhalers has led them to use them erratically only when the symptoms become intolerable.

**Table 3:** Patient information, data, codes and sub-themes for Theme 1 F-female, ID-identity, M-male, yo-year-old

Sub-themes	Codes	Patient ID	Data
Inadequate knowledge	How long to take medicines?	P1 (35-yo F)	How long do I have to take these drugs?” I have already taken it for one year... (investigator clarifies that the patient should continue taking medicines for a lifetime) Oh… I have to take these medicines for my life…. I thought I could take for one year and then stop
	Can I stop after three months?	P8 (22-yo F)	Do I have to take this for my life…..? I am alright most of the time. I get shortness of breath only occasionally….. My symptoms have improved after taking these drugs. Couldn’t I stop the medicine after three months?
Misbelief about the disease and drugs	Mother’s diet	P13 (53-yo F)	I think I got this problem because my mother ate lots of fruits like banana, when I was in her womb; that is why I developed this problem…
	Contagious disease	P18 (37-yo F)	The husband accompanying his wife for her follow-up visit wanted to clarify his doubt…. “Will I catch this disease from her….. She is coughing all the time at home???”
		P9 (64-yo F)	My son doesn’t allow my grandson to come near me because he is small and might catch the disease from me
	I will get addicted to drugs	P3 (45-yo F)	I use them only when I get wheezing. I feel good even without medicines. My relatives tell me not to take medicines unnecessarily as I will get addicted to it

Theme 2: Psychological stress due to disease

The chronic respiratory disease causes disability, impacting the patient's day-to-day life. This disability worsens as the disease progresses. Almost all patients expressed grief about living with their disease condition, especially those from low socioeconomic backgrounds who depend on their daily wages for their living (Table 4). Patients with respiratory problems had to refrain from physical work like carrying heavy loads, bending over, or working continuously for long periods to avoid exacerbation. Since their work expectancy is limited, they are not paid on par with their healthy counterparts. In the worst case, they are not preferred for physically challenging work. Due to their low literacy level, it is difficult to find suitable jobs. Being the head of the family or the only earning family member with asthma or COPD, these patients suffer significant psychological stress due to their inability to support the family financially. A similar situation prevails at home for women who cannot do their daily chores of cooking and cleaning. Women express grief about not being able to do tasks around the house. They had to refrain from dusting, cleaning, or carrying weights. Family members do not understand or support women with asthma at home. These patients have a feeling of worthlessness or helplessness. It is to the extent that one patient was divorced for not being able to wake up early and do the household chores.

**Table 4:** Patient information, data, codes and sub-themes for Theme 2 F-female, ID-identity, M-male, yo-year-old

Sub-themes	Codes	Patient ID	Data
Feeling of worthlessness	Paid less	P32 (46-yo M)	They pay me less compared to other workers... When I go to work… I take rest frequently during my work… I do less work when compared to others...
	Hard to earn a living	P10 (34-yo F)	I cannot clean my house…. I cannot sweep, dust or do any cleaning. I cannot work outside in the fields. I manage to work 7 or 10 days a month for my living
	Not chosen for work	P24 (49-yo M)	The head worker does not choose me because when I go for field work, I cannot work for a long time. When I go for construction work, I cannot do hard jobs like lifting weights. So they don’t choose me. Only when they need more people …..like during harvest, they call me
Feeling of helplessness	Request someone at home to help	P18 (37-yo F)	I cannot use cold water, so I cannot wash my clothes or clean vessels for cooking. I request someone at home to help me with this
	Self-care is not feasible	P14 (41-yo F)	We live in a joint family…. I cannot use hot water for bathing or drinking, and my family members feel it is impossible to do so daily.
	Got divorced	P17 (27-yo F)	I got divorced two months back…..because I cannot come out of my room before 9 o’clock, especially in winter… if I do I will start wheezing… but my in-laws did not understand…
	Cannot start work early	P27 (30-yo F)	I cannot leave my room before 9 o’clock if it’s cold outside. With one breath of cold air, I will start having difficulty breathing.
	Works in a paced manner	P16 (46-yo F)	I used to work very fast. I used to get many incentives while I was working in an export company. My friends used to envy me!!! Now…. I am not going for work. The household chores are challenging. My neighbors and relatives comment sarcastically on my slow pace of work.
	Not informed in-laws before marriage	P22 (20-yo F)	My in-laws tell me I lied and married their son…. They scold me always (cries)… They tell me, when we all are healthy, why are you always falling ill? I drink hot water, bathe in hot water… even then, I get shortness of breath…
No socializing	Travelling is difficult	P9 (64-yo F)	…. travelling is a problem. I stopped visiting my relatives or attending functions…. as I cannot travel. I cannot travel by bus or even by train
Stigma of disease and inhaler use	I don’t want my colleagues to know	P1 (35-yo F)	I don’t carry my inhaler to the workplace. I don’t want my colleagues to know that I have this disease. They may inform the higher officials and I might be expelled from the job.
	I hide my inhaler	P22 (20-yo F)	I hide my inhaler in my bag and use it in toilet so that nobody notices it. They might think I have some dangerous disease
	Friends might tease me	P19 (16-yo M)	I don’t carry my inhaler to school…. I don’t feel comfortable using it in front of my friends. They might tease me….Yes, it happened to one other boy who used an inhaler in school…
		P25 (15-yo M)	I avoid playing games at school…. I am afraid that I will get breathless… I do not want to carry my inhaler… I do not want to use it in school.

CRD patients avoid travelling and visiting family members due to the fear of inducing an exacerbation. Socialization is almost nil in these patients causing psychological stress.

The stigma of using inhalers among friends and colleagues at work or school is common among working people or school-going children. They avoid carrying the inhaler to the workplace, or they would use it in privacy. Using inhalers in public makes them feel like an outcast. Awareness that acute attacks may be triggered at any place leaves the patient insecure and anxious most of the time. Non-availability of reliever drugs in hand during an acute attack might lead to unnecessary hospitalization, which otherwise might have controlled the situation.

Theme 3: Irrelevant lifestyle modifications

All patients with respiratory disease have experienced acute attacks at least once. According to them, it is the most dreadful experience ever. Most patients tearfully voice their feeling of “being close to death” (Table 5). Patients make many adjustments in their day-to-day life to avoid these acute attacks. Elderly patients not only consider these episodes frightful, but it also causes a lot of inconvenience to the family members who have to rush them to a nearby hospital, especially when they occur at odd hours of the day. This gives them a feeling of being a burden to the family.

**Table 5:** Patient information, data, codes and sub-themes for Theme 3 F-female, ID-identity, M-male, yo-year-old

Sub-themes	Codes	Patient ID	Data
Fear of acute attacks	Close to death	P28 (50-yo M)	When I get short of breath… I feel like I am close to death…. It is a horrible experience. I cannot breathe…. I feel like something has caught my throat….
	Terrifying experience	P31 (28-yo M)	I feel something in my throat that’s not allowing me to breathe…It is a terrifying experience… I do not want to experience it again…
Avoid inconvenience to family members	Disturb my son	P30 (49-yo F)	When I get shortness of breath at night, it gets worse… not controlled by my inhalers….becomes unbearable…. I had to disturb my son who lives in a nearby town… he had to take me to the hospital at odd hours…
	I stay alone	P13 (53-yo F)	I stay alone…. My children stay a little far away in a nearby town…. So I try not to fall sick as I do not have anyone to help. I take all precautionary measures. I always use hot water, I avoid foods that are allergic to me… I somehow manage things on my own….
Irrelevant changes in food habits	Does not eat any fruits and avoid some vegetables	P15 (44-yo F)	I do not eat any fruits and I avoid all vegetables like greens, tomatoes, and radish for fear of developing wheezing.
	I feel tired all the time	P25 (15-yo M)	I am very cautious with what I eat. I avoid many vegetables and fruits. I will develop shortness of breath with many of the vegetables... I feel tired all the time because of this….
	I carry boiled water	P24 (49-yo M)	When I go out of town, I carry boiled water in 3 or 4 water bottles. I cannot drink tap water directly as it gives me shortness of breath

The false belief prevails among patients that certain fruits and vegetables trigger acute attacks. They avoid fruits and vegetables like radishes, tomatoes, and greens. These patients also avoided dairy products like curd and buttermilk. Eating unbalanced food makes them feel very weak and lethargic. According to some patients, cold water was also considered a trigger for an acute attack. They carry many bottles of boiled water if they have to stay away from home.

Theme 4: Poor adherence

Being a government-funded tertiary care hospital, JIPMER (Jawaharlal Institute of Postgraduate Medical Education and Research) provides treatment and drugs free of cost to patients. This policy attracts patients belonging to the low-income group to this hospital. Even when medications and inhalers are offered free of charge, other financial reasons were stated to cause poor adherence. The travel expenses and loss of wages incurred for hospital visits are a significant financial burden to these patients. Elderly patients and homemakers, who are financially dependent on family members, find it difficult to come for monthly follow-up visits. They use their drugs sparingly against medical advice, be frugal and only when needed, and come for follow-ups every two or three months, depending on the necessity (Table 6).

**Table 6:** Patient information, data, codes and sub-themes for Theme 4 F-female, ID-identity, M-male, yo-year-old

Sub-themes	Codes	Patient ID	Data
Financial dependence	Take money from my son for my hospital visit	P26 (70-yo M)	I do not take my medicines daily. I take it only when I have a problem with my breath. I cannot visit the hospital every month. I have to take money from my son for my hospital visit… He will ask me a thousand questions before giving me money, so I do not ask him every month. I try to save my medicines for the next one or two months….
	Not able to work full month	P30 (49-yo F)	I am single, and I stay alone. This disease is not allowing me to work an entire month. I work only 10-15 days a month. I earn about 3000 rupees for my living…. How can I spend Rs. 500 every month for my hospital visit? I use my drugs on and off only when needed and try to delay my hospital visits.
Difficulty in travelling	Travel causes shortness of breath	P9 (64-yo F)	I have to travel by bus to go to the hospital…. Travel itself causes shortness of breath, so I avoid travelling… I take my medicines only when needed and save it for the upcoming months.
	Good sleep at night	P2 (41-yo F)	I do not miss the night dose of inhaled medicine and tablet (Deriphylline). I get good sleep after taking these drugs….
Trust in medicine and doctor	Come every month despite the long distance	P5 (40-yo F)	I come every month despite the long distance and money for travel… The medicines given here are very good. I tried buying the same medicines in my town, but that doesn’t work as well as these….

Travelling is another factor that triggers acute attacks in quite a few patients. They tend to compromise on their medication use by using it only when needed and spacing their follow-up visit to once every two to three months. Asthma patients come for regular follow-up visits in winter due to the seasonal worsening of the disease.

Timing of work or school were also stated as a reason for not taking their drugs on time. Going to a job early in the morning or working night shifts were reasons stated by patients for not taking their controller medications regularly.

On the contrary, a patient travelled almost 100 km by bus every month for her follow-up visit accompanied by her husband. She states the contentment of seeing the doctor is the driving force behind her follow-up visit.

Theme 5: Consequence of insufficient monitoring

When patients were questioned regarding their inhaler performance, they voiced that they were initially taught the inhaler technique when they were prescribed. Physicians are not monitoring the inhaler technique during the follow-up visits. When asked to demonstrate, the method has deteriorated over the course of years. Many patients have forgotten the intricate details like exhaling to the residual volume before inhalation, holding breathing after deep inhalation, chin up position during inhalation and gargling after inhalation (Table 7).

**Table 7:** Patient information, data, codes and sub-themes for Theme 5 F-female, ID-identity, M-male, yo-year-old

Sub-themes	Codes	Patient ID	Data
Old age sequela	Inhale from rotacaps directly	P29 (71-yo M)	I sit on the bed and realize that my inhaler machine is not near me… Then I open the capsule and inhale the powder through my mouth…
	Needs help of grandson	P26 (70-yo M)	I cannot load the capsules into the inhaler. I call my grandson to load the capsule… He will help me when he is at home...when he is not around, it is difficult for me
The faded memory of inhaler technique	I do not remember being told to hold my breath or gargle	P12 (59-yo M)	When I first got this machine two years back, I think they told me to keep the capsule in the hole, rotate and then inhale…which I follow till now…
	I do not remember the instructions	P21 (71-yo M)	Yeah… the doctors taught me how to use it… I just inhale forcefully and blow out slowly through the mouth… I do not remember being told to hold my breath or gargle. I drink water after inhaling…
Inappropriate knowledge about drugs	I learnt inhaler technique from a neighbour	P20 (40-yo F)	Doctors did not teach me… I threw the carton box and did not notice the instruction paper inside that box…. I learnt it from a neighbour who also uses a similar machine …
	All or none	P8 (22-yo F)	I take blue and red drugs whenever I have a problem with my breathing; otherwise, I do not use them daily…
	Using reliever regularly	P7 (43-yo F)	Daily I take both blue (salbutamol RC) and red (beclomethasone RC) two times a day. During breathing difficulty, I use both drugs (salbutamol RC and beclomethasone RC) up to five times a day.
	Miss dose due to work	P23 (23-yo M)	I have to leave for work early in the morning… I do not want to take any drugs in an empty stomach in the morning… but I do not miss my nighttime dose.
	Opens one by one	P6 (56-yo F)	I open one bottle, complete it and then open the other bottle. I thought if I broke the seal and opened one bottle, it would spoil if kept for a long time, so I opened one by one. I don’t know if both drugs were different.
		P14 (41-yo F)	I thought both drugs were the same, so I opened one bottle, completed it and then opened the other.

The vital information for a patient with CRD is to know about the different drugs used to treat their condition. They should be aware of when to use the controller and preventer medicines. Most of the study group patients were unaware of this information. They take both these drugs together twice a day or use both sparingly as needed. Some complete one bottle of rotacaps of either salbutamol or beclomethasone and open the next bottle. Some patients report taking beclomethasone in the morning and salbutamol at night or vice versa.

## Discussion

This study disclosed patients’ ignorance of various aspects of the disease. They were unaware of the nature of illness, drugs and inhaler use. The significant findings that affect the patients’ disease control are the erratic use of controller and reliever drugs. Patients executing irrelevant changes in diet and lifestyle to prevent acute exacerbations is another noteworthy fact. A distinguishing fact is the stigmatization of CRD patients at home and in the workplace produces significant psychological and financial stress leading to non-adherence.

Patients not knowing the name of the disease is a common finding in India [7] and other Asian countries like Malaysia [5]. They address it as a “breathing problem”, similar to our study. COPD patients also are unaware of the name of the disease and term it “breathing problem”. Patients were unaware of the disease process leading to frequent exacerbation [7]. Spicy food was thought to trigger asthma symptoms [13], whereas spicy, oily and sour food was thought to trigger COPD exacerbation [7]. Cold weather and dusty environment were also considered asthma triggers in some patients.

In our study, misconceptions prevail among patients and family members regarding the chronic and infectious nature of these diseases. Similar findings were noted in another study from India and other developed countries [14]. Preventing elderly patients from meeting their grandkids leads to social isolation. Inadequate health literacy acts as a barrier to healthcare outcomes. Family members of these patients need to be educated regarding the disease. Precise knowledge about the disease condition is vital in treatment planning and execution. Patients with a poor understanding of the disease have frequent exacerbations [15]. Many studies have proved that health literacy can improve healthcare outcomes [16]. Physicians cannot educate patients due to their limited knowledge [17].

There is a delay in seeking medical care from the onset of symptoms to diagnosis due to unawareness. This study shows an average of seven years of delay in seeking treatment from disease onset. The main reasons are lack of encouragement from family to seek help and lack of insight into the disease condition. Patients seek medical attention only when symptoms impact their lifestyle or reduce their work capacity. This delay in seeking medical attention leads to worsening of the disease.

In this society, females are expected to do cooking, care for their family members and do other household chores. CRD affects the patient's ability to do work. Female patients with CRD cannot keep up with their responsibilities or fulfil the expectations of their family members. This situation causes significant emotional stress to these patients. The feeling of helplessness or worthlessness coupled with no understanding among the family members leads to severe depression and guilt in these patients. Many women in this study group were disheartened regarding this issue. A similar result was seen in Australia, where asking for help from family members, difficulty in cooking and having to stay indoors during winter had a significant impact emotionally among COPD patients in Australia [18]. A study showed hesitancy in marrying a person with asthma to prevent passing the disease to the next generation. Asians living in the USA consider asthma unacceptable in Asian society and hence do not reveal their illness to others [13]. A female patient in this study was divorced due to her inability to do household chores.

Acute attacks are a significant threat among patients with respiratory disease. The experience of an acute attack makes patients feel asthma is a life-threatening condition. COPD patients think they live at the mercy of the disease [11]. Many vegetables and fruits were believed to precipitate an acute attack. Patients avoid the consumption of these items, which are rich in vitamins and minerals. This results in patients eating an unbalanced diet. The nutritional status of these patients tends to decline due to their improper dietary intake. By avoiding these diets, the patients do not get the essential nutrients and tend to become malnourished. A review on nutritional screening in COPD patients revealed that these patients have a tendency to become malnourished and should consume a lot of vegetables, fruits and milk. Patients consuming foods rich in vitamins A, D and E and pigmented vegetables have improved lung function [19].

Limiting physical activity is another method to prevent acute attacks. To accommodate themselves to daily life, the patients change how they work. They stay indoors, do chores slowly, and avoid bending, dusting, sweeping or contact with cold water. Some patients experience a loss of independence or decreased self-esteem, or loss of financial independence, which disrupts their family life. All these factors cause significant psychological stress.

Despite providing free treatment and medications, this study has shown that cost of travelling and loss of wage for the hospital visit poses a significant financial burden. This has led the patients to use their drugs sparingly despite being aware of their proper use. Patients with chronic disease stop medications when they are symptom free. Erratic use of reliever and controller drugs either due to misunderstanding or financial reasons was noted. Patients need to know the importance of taking their medications regularly.

In this community, patients believe that drugs used for treating CRD are addictive or develop tolerance with time. Another study reported similar beliefs along with other negative thoughts, like the development of cancer or organ failure. These negative beliefs impair disease control due to nonadherence [20]. Patients are ambivalent about the use of steroids. Some feel inhaled corticosteroids (ICS) does not work and stop using steroid inhalers, which was also noted in this study. Most patients prefer oral medications compared to inhalers. Besides forgetting and inconvenience with using the inhaler, lousy taste and side effects were other reasons for not using inhalers. Elderly patients have poor inhaler technique due to cognitive impairment, physical disability and poor coordination associated with age. This study revealed that elderly patients needed help from their family members to insert the rotacap in the capsule holder in the Rotahaler. Side effects are the major obstacle in the treatment of the elderly [11]. Generally, COPD patients are older and have difficulty handling either metered-dose inhalers (MDI) or dry powder inhalers (DPI) [21]. COPD patients have a low level of intentional nonadherence [10]. Continuous reinforcement improves patient knowledge and self-management behaviours, consecutively improving treatment outcomes and quality of life [22].

The stigma in revealing the disease condition to friends and family was reported in many studies [14,16]. Our research noted stigma in revealing the disease to co-workers and using the inhaler at work. Patients do not discuss their health condition with co-workers or higher-ups at the workplace for fear of being expelled from work which would affect them financially [23]. The stigma of using the inhaler at work has been an issue according to many studies [13,24]. An Indian study revealed that an inhaler was not the preferred route of drug delivery for asthma patients. There was inhibition in using inhalers in public or in front of their friends [24]. Patients consider productivity at work more important than their health and safety [23]. Employers do not employ asthma patients as they are likely to take a long leave of absence [14]. Stigmatization is another reason that leads patients to seek delayed medical advice and disrupts their everyday social life [5]. Healthcare providers should provide at least vital information to patients to ensure the appropriate use of the drugs. Prescribing the drugs according to the patient's symptoms will not complete the treatment. Healthcare providers should ensure the proper use of medicines [25].

Even though this study's qualitative design helped bring out the concerns and views about CRD and its treatment in this population, the discrepancy in the number of asthma and COPD patients and the number of male and female patients was a limitation of this study. The data collected was not analyzed separately for asthma and COPD subgroups or different levels of severity.

## Conclusions

This study sheds light on patients’ understanding and knowledge about the disease and its impact on disease management. Subjective reporting by patients in this study was helpful in understanding issues concerning disease management in CRD patients. Asthma management studies have shown to reduce acute attacks, hospital admissions, days off work, and improved symptoms and inhaler technique. Apart from assessing the patients’ symptoms and prescribing drugs, healthcare providers should take time to impart knowledge about the disease to patients. Though patient education and psychological intervention are challenging to implement daily, they are supplemental to pharmacologic management of the disease. Patients’ underlying beliefs about the illness must be understood to change their behavior towards their condition. Patients must be detailed about the cause of the disease and should be provided action plans to tackle acute attacks to avoid panic during exacerbations. Family members of patients should also be educated appropriately. Healthcare professionals should work on removing the stigma about the disease and drugs. The use of inhalers should be reviewed during their follow-up visits, and correct technique should be ensured. Involving the patient in decision-making and keeping in mind their emotional, physical and family status while prescribing medicines would improve adherence.
